# Phytochemical investigation and nephroprotective potential of *Sida cordata* in rat

**DOI:** 10.1186/s12906-017-1896-8

**Published:** 2017-08-04

**Authors:** Naseer Ali Shah, Muhammad Rashid Khan, Dereje Nigussie

**Affiliations:** 1 Department of Biosciences, COMSATS Institute of Information Technology, Islamabad, Pakistan; 2 Department of Biochemistry, Faculty of Biological Sciences, Quaid-i-Azam University, Islamabad, , 45320 Pakistan; 3 Ethiopian Public Health Institute, Addis Ababa, Ethiopia

**Keywords:** *S. cordata*, Renal function test, Antioxidant enzymes, Cellular injuries, Blood, Ethyl acetate

## Abstract

**Background:**

Plants are an efficient source of natural antioxidant against free radicals causing kidney damages. *Sida cordata* ethyl acetate fraction has been reported for strong in vitro antioxidant potency, previously. In the present study, our objective was to evaluate its in vivo antioxidant potency against CCl_4_ induced nephrotoxicity and investigates the bioactive phytochemicals by HPLC-DAD analysis.

**Methods:**

Phytochemical analysis was performed by HPLC-DAD methodology. For in vivo study, 42 male Sprague-Dawley rats were treated with alternatively managed doses for 60 days. Group I animals were remained untreated. Group II animals were treated with vehicle (1 mL of olive oil) by intragastric route on alternate days. Group III was treated with 30% CCl_4_ (1 mL/kg b.w.) i.p. Group IV was treated with 30% CCl_4_ (1 mL/kg b.w.) i.p and silymarin intragastric. Group V and VI rats were treated with 30% CCl_4_ and SCEE (150 and 300 mg/kg b.w., respectively) intragastric. Group VII animals were treated with SCEE (300 mg/kg b.w.) intragastrically. Blood parameters, Serum proteins and urine profile were investigated. Activities of tissue enzyme i.e. catalase, peroxidase, superoxide dismutase, glutathione-S-transferase, glutathione reductase, GSH and γ-GT were evaluated. Histopathological observations, total protein contents, lipid peroxidation, DNA damage and relative weight were also analyzed.

**Results:**

Gallic acid, catechin and caffeic acid were identified in SCEE fraction by HPLC-DAD. Decrease in the count of red blood cells, neutrophils, eosinophils and concentration of hemoglobin whereas increase in lymphocyte count and estimation of sedimentation rate (ESR) with 1 mL CCl_4_ (30% in Olive oil) administration (30 doses in 60 days) was restored dose dependently with co-treatment of SCEE (150 and 300 mg/kg b.w.). Treatment of rats with CCl_4_ markedly (*P* < 0.01) increased the count of urinary red blood cells and leucocytes, concentration of urea, creatinine and urobilinogen and specific gravity whereas creatinine clearance was reduced. Serum level of total protein, albumin, globulin, nitrite, creatinine and blood urea nitrogen (BUN) was significantly increased (*P* < 0.01) by CCl_4_ treatment. The activity of antioxidant enzymes; catalase, superoxide dismutase, glutathione peroxidase, glutathione-S-transferase and glutathione reductase and content of reduced glutathione was decreased (*P* < 0.01) significantly. However, increased concentration (*P* < 0.01) of thiobarbituric acid reactive substances and histopathological injuries were noticed in the renal tissues of rats after the treatment with CCl_4_. Co-administration of SCEE, dose dependently, protected the alterations in the studied parameters of rats at 150 and 300 mg/kg b.w. The present study revealed that SCEE could be used as a possible remedy for renal toxicity abnormalities.

**Conclusion:**

These results are an evidence of the renal protective role of *S.cordat* ethyl acetate fraction against CCl_4_ induced nephrotoxicity in rats which may be due to its antioxidant compounds.

## Background

Carbon tetrachloride (CCl_4_) is a well-known agent that is commonly used in the dry-cleaning industry and has been proven to have a highly hepatotoxic as well as nephrotoxic effect. CCl_4_ can lead to acute tubular necrosis in the kidney which leads to cirrhosis [1–5]. Its harmful effect on the kidney occurs due to the CCl_4_ toxic metabolites; trichloromethyl and trichloromethylperoxy radicals inherent in the cytochrome P450 system. These reactive species trigger lipid peroxidation along with reduced total proteins [6] which are then controlled through antioxidant enzymes e.g. catalase, peroxidase and superoxide dismutase and phase II metabolizing enzymes which prevent lipid peroxidation in the kidney [7, 8]. Due to its well explained mechanism of toxicity induction, many studies have adopted it as renal free radicals inducing agent [9–13].

To cope with such reactive species, it is essential to obtain dietary antioxidants to counteract the excess of these species**.** Plant-derived natural antioxidants are proven to be very effective to control toxicities and stressed conditions triggered by CCl_4_ radicals in renal injuries [5, 14–19]. Plants secondary metabolites i.e. polyphenols can scavenge free radicals and control the oxidative damage of proteins and lipids [20].

*Sida cordata*, a member of the Malvaceae Family, is a trailing wayside herb found frequently growing in shady places. It is cosmopolitan in Pakistan, India and other tropical countries and is mostly distributed in the forest and as weeds in the overgrown grass of gardens and public parks [21]. It is known as Faridbuti, Rajbala, Bhumibala and Shaktibala in India and Simak in Pakistan. It is extensively used for therapeutic purposes in the codified Indian systems of medicine namely Siddha and Ayurveda. Its roots are used as diuretic, astringent, stomachic, febrifuge and demulcent and seeds are applied as a laxative, aphrodisiac and demulcent and recommended in cystitis, colic, gonorrhea, tenseness and piles. In folk medicine, female uses it as soup in the last days of pregnancy to reduce the pain of labor and reducing the period [22]. In our preliminary study, we have reported in vitro antioxidant potential of *S. cordata* by using different antioxidant assays such as DPPH, H_2_O_2_, NO^−^ and anti-lipid peroxidation assays. Amongst extract and fractions studied, ethyl acetate was observed to be the most potent one in all assays [22]. Therefore, this study was designed to investigate its protective effect against CCl_4_ induced toxicity on kidneys of the rat.

## Methods

### Chemicals

Reduced glutathione (GSH), oxidized glutathione (GSSG), glutathione reductase, γ-glutamyl*p*-nitroanilide, glycylglycine, bovine serum albumin (BSA), 1,2-dithio-bis nitro benzoic acid (DTNB), 1-chloro-2,4-dinitrobenzene (CDNB), reduced nicotinamide adenine dinucleotide phosphate (NADPH), flavine adenine dinucleotide (FAD), glucose-6-phosphate, Tween-20, 2,6-dichlorophenolindophenol, thiobarbituric acid (TBA), picric acid, sodium tungstate, sodium hydroxide, trichloroacetic acid (TCA) and perchloric acid (PCA) were purchased from Sigma Chemicals Co. St. Louis, USA.

#### Plant collection

The whole plant was collected from the campus of Quaid-i-Azam University, Islamabad, Pakistan and recognized by their local names and then confirmed by Prof. Dr. Mir Ajab Khan, Department of Plant Sciences, Quaid-i-Azam University, Islamabad. Voucher specimen with accession No. 27824 was deposited at the Herbarium, Quaid-i-Azam University, Islamabad.

Shade dried 4 Kg powder of *S. cordata* whole plant was extracted twice for 72 h in 8 l of methanol and filtered through Whatman filter paper # 45, and the filtrate was concentrated through rotary vacuum evaporator to get the crude methanol extract and its yield was 8%. To fractionate the extract in increasing order of polarity, it was suspended in distilled water (6 g/250 mL) and passed through different solvents (250 mL each) in the order of *n*-hexane → ethylacetate → *n*-butanol to get different fractions by using separating funnel. Non polar *n*-hexane yield was 35% of dry methanol extract, while polar ethyl acetate and *n*-butanol yield was 15 and 10% of dry methanol extract, respectively. Residue fraction known as aqueous fraction gave a yield of 40%. All the fractions were stored at 4 °C until further use.

#### HPLC analysis

Chromatographic analysis was carried out by using HPLC-DAD attached with Agilent RP-C8 analytical column. Briefly, mobile phase A was acetonitrile-methanol-water-acetic acid (5:10:85:1) and mobile phase B was acetonitrile- methanol- acetic acid (40:60:1). A gradient of time 0–20 min for 0 to 50% B, 20-25 min for 50–100% B and then isocratic 100% B till 40 min was used. The flow rate was 1 mL/min and the injection volume was 20 μL. All the samples were analyzed at 257, 279, 325 and 368 nm wavelength. The column was reconditioned for 10 min before the next analysis. All samples were assayed in triplicate. Standards (catechin, rutin, kaempferol, quercetin, gallic acid, salicylic acid, apigenin, myricetin and caffeic acid (Sigma company, USA) and plant extract stock solutions were prepared in methanol at a concentration of 200 μg/mL and 10 mg/mL, respectively. Samples were filtered through 0.45 μm membrane filter. Quantification was carried out by the integration of the peak using the external standard method. All chromatographic operations were carried out at ambient temperature.

#### Animals and treatment

Six-week-old 42 male Sprague-Dawley rats weighing 190 ± 10 g were provided with food and water ad libitum and kept at 23–25 °C on a 12-h light-dark cycle. All experimental procedures involving animals were conducted in accordance with the guidelines of National Institutes of Health (NIH guidelines Islamabad, Pakistan). The study protocol was approved by Ethical Committee of Quaid-i-Azam University, Islamabad, Pakistan. The rats were acclimatized to laboratory condition for 7 days before commencement of the experiment. The following experimental groups (06 rats per group) were studied.

Group I animals were remained untreated. Group II animals were treated with vehicle; 1 mL of olive oil by intragastric route on alternate days (30 doses) for 60 days. Group III was treated with 30% CCl_4_ (1 mL/kg b.w.) i.p on an alternate day for 60 days. Group IV was treated with 30% CCl_4_ (1 mL/kg b.w.) i.p and silymarin intragastric on an alternate day for 60 days. Groups V and VI rats were treated with 30% CCl_4_ and SCEE (150 and 300 mg/kg b.w., respectively) intragastric on an alternate day for 60 days. Group VII animals were treated with SCEE (300 mg/kg b.w.) intragastrically on alternate days for 60 days.

After completion of the dosages, rats were kept individually in metabolic cages for 24 h; collected the urine and volume was determined. All the animals were sacrificed and blood was drawn prior to the excision of the organ. The serum was stored at −80 °C until it was assayed as described below. Half of kidney tissues were treated with liquid nitrogen and stored at −80 °C for further enzymatic analysis while the other portion was processed for histological examination.

### Analysis of urine

Urine samples were assayed for pH, specific gravity, urea, creatinine, protein, albumin, urobilinogen, red blood cells (RBCs) and white blood cells (WBCs) count by using standard diagnostic kits (MediScreen Urine Strips, Orgenics, France) and standard AMP diagnostic kits (StattoggerStrasse 31b 8045 Graz, Austria). Urinary creatinine clearance was estimated by using the formula:

CrCl = U × V/P × T Where.

U: concentration of creatinine in urine, P: concentration of creatinine in plasma, V: 24 h of urinary volume, T: Time in minutes.

### Analysis of serum

Analysis of serum for blood urea nitrogen (BUN), nitrite, creatinine, total protein, and albumin was estimated by using standard AMP diagnostic kits (Stattogger Strasse 31b 8045 Graz, Austria).

### Assessment of antioxidant enzymes profile

Renal tissues were homogenized in 10 volume of 100 mM KH_2_PO_4_ buffer containing 1 mM EDTA (pH 7.4) and centrifuged at 12,000×g for 30 min at 4 °C. The supernatant was collected and used for enzymatic studies. The protein concentration of kidney tissue supernatant was determined using crystalline BSA as standard [23].

#### Catalase assay (CAT)

CAT activity was determined by the method of NA Shah, MR Khan, B Ahmad, F Noureen, U Rashid and RA Khan [22]. The reaction solution of CAT activity contained: 2.5 mL of 50 mM phosphate buffer (pH 5.0), 0.4 mL of 5.9 mM H_2_O_2_ and 0.1 mL enzyme homogenate. Changes in absorbance of the reaction solution were determined after one min at 240 nm. One unit of CAT activity was defined as an absorbance change of 0.01 as units/min.

### Peroxidase (POD) activity

B Chance and A Maehly [24] methodology was used to determine POD activity spectrophotometrically with minor modifications. To 25 μL of tissue homogenate, 25 μL of 20 mM guaiacol, 75 μL of 40 mM H_2_O_2_ and 625 μL of 50 mM potassium phosphate buffer (pH 5.0) was added. Absorbance change was measured at 470 nm. Change in absorbance of 0.01 as units/min defines one unit POD activity.

### Superoxide dismutase assay (SOD)

SOD activity of kidney tissues was estimated by the method of NA Shah, MR Khan, B Ahmad, F Noureen, U Rashid and RA Khan [22]. Reaction mixture of this method contained: 0.1 mL of phenazine methosulphate (186 μM), 1.2 mL of sodium pyrophosphate buffer (0.052 mM, pH 7.0), 0.3 mL of supernatant after centrifugation (1500×g for 10 min followed by 10,000×g for 15 min) of kidney homogenate was added to the reaction mixture. The enzyme reaction was initiated by adding 0.2 mL of NADH (780 μM) and stopped after 1 min by adding 1 mL of glacial acetic acid. The amount of chromogen formed was measured by recording color intensity at 560 nm. Results are expressed in units/mg protein.

### Glutathione-S-transferase assay (GST)

The reaction mixture of glutathione-S-transferase activity consisted of 1.475 mL phosphate buffer (0.1 M, pH 6.5), 0.2 mL reduced glutathione (1 mM), 0.025 mL (CDNB; 1 mM) and 0.3 mL of tissue homogenate in a total volume of 2 mL. The changes in the absorbance were recorded at 340 nm and enzymes activity was calculated as nM CDNB conjugate formed/min/mg protein using a molar extinction coefficient of 9.6 × 10^3^ M^−1^ cm^−1^ [25].

### Glutathione reductase assay (GSR)

Glutathione reductase activity was determined by following the protocol of RA Khan, MR Khan, S Sahreen and J Bokhari [25]. The reaction mixture consisted of 1.65 mL phosphate buffer: (0.1 M; pH 7.6), 0.1 mL EDTA (0.5 mM), 0.05 mL oxidized glutathione (1 mM), 0.1 mM NADPH (0.1 mM) and 0.1 mL of homogenate in a total volume of 2 mL. Enzyme activity was quantified at 25 °C by measuring disappearance of NADPH at 340 nm and was calculated as nM NADPH oxidized/min/mg protein using molar extinction coefficient of 6.22 × 10^3^ M^−1^ cm^−1^.

### Glutathione peroxidase assay (GPx)

Glutathione peroxidase activity was assayed by the method of NA Shah, MR Khan, B Ahmad, F Noureen, U Rashid and RA Khan [22]. The reaction mixture consisted of 1.49 mL phosphate buffer (0.1 M; pH 7.4), 0.1 mL EDTA (1 mM), 0.1 mL sodium azide (1 mM), 0.05 mL glutathione reductase (1 IU/mL), 0.05 mL GSH (1 mM), 0.1 mL NADPH (0.2 mM), 0.01 mL H_2_O_2_ (0.25 mM) and 0.1 mL of homogenate in a total volume of 2 mL. The disappearance of NADPH at 340 nm was recorded at 25 °C. Enzyme activity was calculated as nM NADPH oxidized/min/mg protein using molar extinction coefficient of 6.22 × 10^3^ M^−1^ cm^−1^.

### Reduced glutathione assay (GSH)

1.0 mL homogenate was precipitated with 1.0 mL of (4%) sulfosalicylic acid. The samples were kept at 4 °C for 1 hour and then centrifuged at 1200×g for 20 min at 4 °C. The total volume of 3 mL assay mixture contained: 0.1 mL filtered aliquot, 2.7 mL phosphate buffer (0.1 M; pH 7.4) and 0.2 mL DTNB (100 mM). The yellow color developed was read immediately at 412 nm on a SmartSpecTM plus Spectrophotometer. It was expressed as μM GSH/g tissue [22].

#### Estimation of lipid peroxidation (TBARS)

The assay for lipid peroxidation was carried out with modified method of RA Khan, MR Khan and S Sahreen [26]. The reaction mixture in a total volume of 1 mL contained: 0.8 mL phosphate buffer (0.1 M, pH 7.4) and 0.2 mL homogenate sample. The reaction mixture was incubated at 37 °C in a shaking water bath for one hour. The reaction was stopped by addition of 1.0 mL 10% trichloroacetic acid following addition of 1 mL 0.67% thiobarbituric acid. All the tubes were placed in boiling water bath for 20 min and then shifted to crushed ice-bath before centrifuging at 2500×g for 10 min. The amount of malonaldehyde formed in each of the samples was assessed by measuring the optical density of the supernatant at 535 nm using spectrophotometer against a reagent blank. The results were expressed as nM of TBARS/min/mg tissue protein.

#### Nitrite/nitrate assay

Nitrite/nitrate was assayed colorimetrically in tissue homogenate by using the methodology of R Berkels, S Purol-Schnabel and R Roesen [27]. Promega’s Griess reagent system is based on the chemical reaction between sulfanilamide and N-1-naphthylethylenediamine dihydrochloride under acidic condition (phosphoric acid) to give bright reddish-purple colored azo-compound which can be measured at 540 nm spectrophotometrically. Using standard curve of sodium nitrite, nitrite concentration in tissue samples was calculated.

#### H_2_O_2_ assay

The methodology of E Pick and Y Keisari [28] was adopted to determine the H_2_O_2_-mediated horseradish peroxidase-dependent oxidation of phenol red. An aliquot of 100 μL of tissue homogenate was added to 100 μL of 0.28 nM phenol red, 250 μL of 5.5 nM dextrose, 8 units of horseradish peroxidase and 500 μL of 0.05 M phosphate buffer (pH 7.0) and incubated at room temperature for 1 hour. The reaction was stopped by the addition of 100 μL of 10 N NaOH and then tubes were centrifuged for 10 min at 800×g. The supernatant was collected and absorbance was measured at 610 nm using reagent as blank. The quantity of H_2_O_2_ produced was expressed as nM H_2_O_2_/min/mg tissue based on the standard curve of H_2_O_2_ oxidized phenol red.

#### Tissue protein estimation

The total amount of soluble proteins in tissue homogenate was determined by the method of OH Lowry, NJ Rosebrough, AL Farr and RJ Randall [29]. To the tissue homogenate, 300 μL of 0.1 M potassium phosphate buffer (pH 7.0) was added in order to dilute the tissue sample. To this mixture, 1 mL of alkaline copper solution was added and kept at room temperature. After 10 min of incubation, 100 μL of Folin-Ciocalteau phenol reagent was added. Reaction tubes containing test mixture was then vortexed and again incubated 37 °C for 30 min. Optical density was measured spectrophotometrically at 650 nm. Total soluble proteins of tissue samples were then determined using the standard curve of bovine serum albumin.

#### Histopathological determination

For microscopic evaluation, tissues were fixed in a fixative (absolute ethanol 60%, formaldehyde 30% and glacial acetic acid 10%) and embedded in paraffin, sectioned at 4 μm and subsequently stained with hematoxylin/eosin. Sections were studied under a light microscope (DIALUX 20 EB) at 40 magnifications. Slides of all the treated groups were studied and photographed. A minimum 12 fields of each section were studied and approved by a pathologist without knowing its treatment nature.

#### Statistical analysis

The values were expressed as the mean ± SD for the 06 rats in each group. Differences between groups were assessed by one-way analysis of variance (ANOVA) with Statistix 8.1. A value corresponding to *P* < 0.05 was deemed to be statistically significant.

## Results and discussion

Antioxidants play important role in reduction of chronic diseases like DNA damage, mutagenesis and carcinogenesis, and inhibition of pathogenic bacteria growth [30].

HPLC analysis for the presence of flavonoids and phenolics in SCEE fraction expressed gallic acid, catechin and caffeic acid. Caffeic acid was observed in maximum quantity (11.42 ± 0.90 μg/mg dry sample) (Table 1). Diverse biological activities for these compounds have been reported. Such as, neuroprotective, anti-apoptotic and anti-inflammatory in clinical disorders for catechin [31], anti-metastatic and anti-tumour activity for caffeic acid [32] and prevention of rancidity induced by lipid peroxidation and spoilage for gallic acid [33].

**Table 1:** HPLC profile of Ethyl acetate fraction of *S. cordata*

Flavonoid/Phenolics	Signal wavelength	Retention time (min)	Quantity (μg/mg dry sample)
Gallic acid	257	4.593	1.81 ± 0.15
Catechin	279	7.334	1.26 ± 0.01
Caffeic acid	325	9.66	11.42 ± 0.90

For in vivo study of SCEE, doses of 150 and 300 mg/kg b.w. were selected from NA Shah, MR Khan, B Ahmad, F Noureen, U Rashid and RA Khan [22] previous reports. Effect of CCl_4_ treatment and preventive role of SCEE on RBC, Hb and ESR is shown in Table 2. CCl_4_ administration caused a significant reduction in the count of RBCs and Hb and an increase in ESR. Co-administration of SCEE prevented alteration in the count of RBCs and in the concentration of Hb and ESR, dose dependently. At 300 mg/kg b.w. dose, the protective effect of SCEE against CCl_4_ induced toxicity in respect to RBC, Hb and ESR was in parallel to that of the silymarin co-treated group. CCl_4_ treatment significantly reduced the number of total leukocyte count and differential leukocyte count (Table 3). Total leukocyte count was significantly reversed by SCEE co-treatment. In differential count, a number of monocytes and eosinophils remained uniform in all groups and no significant alteration was observed. Count of neutrophils and lymphocytes showed alteration in peripheral blood. The number of neutrophils was decreased significantly from 59.6 ± 1.5 to 44.3 ± 0.6% which was reversed by SCEE dose dependently. At the higher dose, SCEE showed equal protection to that of the silymarin co-treated group. Lymphocytes percentage was increased from 35.6 ± 1.5 to 52.0 ± 1.0%. This change was prevented by SCEE dose dependently. The reduction of hematological parameters in CCl_4_ administered groups was enhanced in the animal co-treated with the SCEE and this was comparative to the effects acquired by I Meral and M Kanter [34], who reported an enhanced level of hematological parameters in rats treated with *Nigella sativa* against CCl_4_ harmfulness. This change in hematological parameters could be attributed to its bioactive constituents of the antioxidant fraction.

**Table 2:** Effect of different treatments of *S. cordata* “ethyl acetate fraction (SCEE)” on RBC, Hb and ESR profile

Group	RBC(10^6^ /mm^3^)	Hb(g/dl)	ESR
Control	5.00 ± 0.10^ab^	8.83 ± 0.28^a^	9.16 ± 0.76^c^
Vehicle control	4.86 ± 0.20^b^	8.50 ± 0.43^a^	9.00 ± 1.00^c^
CCl_4_ (1 mL/kg)	4.00 ± 0.10^d^	6.16 ± 0.22^c^	20.2 ± 0.75^a^
Silymarin + CCl_4_	4.90 ± 0.15^b^	8.30 ± 0.62^ab^	10.1 ± 0.76^c^
SCEE (150 mg/kg) + CCl_4_	4.40 ± 0.12^c^	7.23 ± 0.25^bc^	13.4 ± 0.66^b^
SCEE (300 mg/kg) + CCl_4_	4.90 ± 0.15^b^	8.66 ± 0.64^a^	10.7 ± 0.46^c^
SCEE (300 mg/kg)	5.30 ± 0.10^a^	9.20 ± 0.34^a^	9.66 ± 0.57^c^

Values expressed as means ± SD. Means ± SD with different superscript letter within the column indicate significant difference (*P* < 0.05)

**Table 3:** Effect of different treatments of *S. cordata* “ethyl acetate fraction (SCEE)” on total leucocytes count and differential leukocytes count

Group	Total Leukocytes count	Differential Leukocyte Count (%)			
		Neutrophils	Lymphocytes	Oesinophils	Monocytes
Control	7450.0 ± 132.2^a^	59.6 ± 1.5^a^	35.6 ± 1.5^c^	3.33 ± 0.57^a^	1.33 ± 0.57^a^
Vehicle control	7683.3 ± 125.8^a^	59.0 ± 4.6^a^	35.6 ± 3.5^c^	3.66 ± 1.15^a^	1.66 ± 0.57^a^
CCl_4_ (1 mL/kg)	5733.3 ± 251.6^c^	44.3 ± 0.6^c^	52.0 ± 1.0^a^	2.33 ± 0.59^a^	1.66 ± 0.57^a^
Silymarin + CCl_4_	7300.0 ± 200.0^a^	60.6 ± 0.6^a^	34.0 ± 1.7^c^	3.66 ± 0.60^a^	1.66 ± 0.57^a^
SCEE (150 mg/kg) + CCl_4_	6400.0 ± 100.0^b^	52.3 ± 2.1^b^	43.0 ± 1.0^b^	3.00 ± 1.00^a^	1.66 ± 0.57^a^
SCEE (300 mg/kg) + CCl_4_	7600.0 ± 200.0^a^	61.0 ± 1.0^a^	36.0 ± 1.0^c^	2.00 ± 0.00^a^	1.33 ± 0.57^a^
SCEE (300 mg/kg)	7716.7 ± 175.5^a^	61.0 ± 1.7^a^	35.0 ± 1.0^c^	2.66 ± 0.53^a^	1.33 ± 0.57^a^

Values expressed as means ± SD. Means ± SD with different superscript letter within the column indicate significant difference (*P* < 0.05)

Urinalysis could possibly give data in regards to the status of kidney and liver functional capacity and acid-base balance [35]. Urine profile was used to assess the kidney function of animals used in the experiment against oxidative stress induced by the CCl_4_ intoxication. The urine pH was decreased significantly after administration of CCl_4_ in comparison to that of the control group, which was protected in the SCEE co-treated groups. The specific gravity of urine and the number of RBC, WBC and urea was noted significantly higher in CCl_4_ treated groups than the control groups. A dose-dependent protection to that of the control group was observed in SCEE co-administered groups (Table 4).

**Table 4:** Effect of different treatments of *S. cordata*“ethyl acetate fraction (SCEE)” on urine pH, S. gravity, RBC, WBC and urea profile

Group	pH	Specific gravity	RBC/μl	WBC/μl	Urea (mg/dl)
Control	7.05 ± 0.04^a^	1.02 ± 0.01^c^	0.01 ± 0.00^d^	14.0 ± 1.7^c^	65.6 ± 1.2^c^
Vehicle control	7.07 ± 0.01^a^	1.03 ± 0.02^c^	0.00 ± 0.00^d^	15.6 ± 4.0^c^	65.3 ± 1.5^c^
CCl_4_ (1 mL/kg)	6.00 ± 0.20^b^	1.48 ± .040^a^	12.0 ± 1.73^a^	67.6 ± 6.8^a^	95.6 ± 3.1^a^
Silymarin + CCl_4_	7.06 ± 0.04^a^	1.02 ± 0.01^c^	0.66 ± 0.57^c^	17.3 ± 6.8^c^	62.0 ± 3.0^c^
SCEE (150 mg/kg) + CCl_4_	6.92 ± 0.28^a^	1.22 ± .030^b^	8.00 ± 1.00^b^	48.3 ± 3.5^b^	79.3 ± 1.5^b^
SCEE (300 mg/kg) + CCl_4_	7.12 ± 0.06^a^	1.02 ± 0.02^c^	0.03 ± 0.00^d^	18.6 ± 6.1^c^	67.0 ± 2.0^c^
SCEE (300 mg/kg)	7.12 ± 0.15^a^	1.02 ± 0.02^c^	0.01 ± 0.01^d^	14.6 ± 2.5^c^	64.6 ± 1.2^c^

Values expressed as means ± SD. Means ± SD with different superscript letter within the column indicate significant difference (*P* < 0.05)

Creatinine clearance, albumin/creatinine ratio**,** urobilinogen and urinary protein levels were estimated in different groups and are given in Table 5. Creatinine clearance decreased after CCl_4_ administration but SCEE co-administration raised it significantly but its value remained significantly lower when compared to that of the control group. The level of urinary protein, albumin/creatinine ratio and urobilinogen also showed alteration after CCl_4_ treatment and a significant decrease was observed. The restoration was observed dose dependently in groups co-treated with SCEE.

**Table 5:** Effect of different treatments of *S. cordata*“ethyl acetate fraction (SCEE)” on urine creatinine, creatinine clearance, albumin, urobilinogen and protein profile

Group	Albumin/ Creatinine ratio (mg/dL)	Creatinine clearance (mLmin)	Urinary protein (mg/dL)	Urobilinogen (mg/dL)
Control	6.3^a^	1.20 ± 0.02^a^	41.6 ± 1.5^a^	2.03 ± 0.15^c^
Vehicle control	6.3^a^	1.18 ± 0.04^ab^	40.0 ± 2.0^a^	2.13 ± 0.20^c^
CCl_4_ (1 mL/kg)	2.0^d^	0.68 ± 0.02^e^	18.6 ± 1.6^d^	23.0 ± 2.00^a^
Silymarin + CCl_4_	5.0^b^	1.11 ± 0.01^b^	33.0 ± 1.0^b^	2.40 ± 0.52^c^
SCEE (150 mg/kg) + CCl_4_	3.4^c^	0.78 ± 0.04^d^	24.3 ± 1.5^c^	11.0 ± 1.00^b^
SCEE (300 mg/kg) + CCl_4_	4.3^b^	0.92 ± 0.02^c^	32.3 ± 1.6^b^	4.50 ± 0.50^c^
SCEE (300 mg/kg)	6.6^a^	1.20 ± 0.02^a^	42.0 ± 1.0^a^	2.10 ± 0.17^c^

Values expressed as means ± SD. Means ± SD with different superscript letter within the column indicate significant difference (*P* < 0.05)

Serum profile can also be used to assess proper functioning of a urinary system like urine profile. Serum profile of kidney function test is shown in Table 6. CCl_4_ treatment significantly lowered protein and albumin content in comparison to that of control group and negative control group. SCEE treatment dose-dependently erased the effect of CCl_4_ intoxication and significantly improved protein and albumin content. SCEE at 300 mg/kgb.w. dose showed an equal level of protection of protein and albumin to that of reference drug silymarin treated group. BUN, creatinine and nitrite content profile also changed after CCl_4_ treatment and values were raised significantly after CCl_4_ treatment. Silymarin and SCEE treatment significantly reversed the increased profile near to that of control group level, showing protection against CCl_4_ toxicity. No significant change was observed in serum profile by oral administration of SCEE (300 mg/kg bw) in comparison to that of control and negative control group. Prior studies have also indicated that different plant extracts extensively improved the renal damages impelled through CCl_4_ intoxication [17].

**Table 6:** Effect of different treatments of *S. cordata*“ethyl acetate fraction (SCEE)” on serum protein, albumin, BUN, creatinine and nitrite profile

Group	Serum proteins (mg/dL)	Albumin (mg/dL)	BUN (mg/dL)	Creatinine (mg/dL)	Serum nitrite (μM/mL)
Control	93.0 ± 2.6^a^	52.3 ± 3.2^a^	45.6 ± 1.2^c^	3.33 ± 0.28^d^	36.0 ± 2.6^c^
Vehicle control	91.0 ± 1.0^a^	49.0 ± 2.0^a^	45.3 ± 1.5^c^	3.91 ± 0.10^cd^	39.6 ± 0.6^c^
CCl_4_ (1 mL/kg)	39.6 ± 1.5^d^	16.0 ± 2.6^c^	75.6 ± 3.1^a^	6.91 ± 0.10^a^	73.6 ± 2.1^a^
Silymarin + CCl_4_	84.0 ± 3.6^b^	39.3 ± 2.5^b^	42.0 ± 3.0^c^	4.43 ± 0.40^c^	28.0 ± 1.0^d^
SCEE (150 mg/kg) + CCl_4_	61.6 ± 1.5^c^	20.3 ± 2.1^c^	59.3 ± 1.5^b^	5.46 ± 0.32^b^	58.3 ± 1.5^b^
SCEE (300 mg/kg) + CCl_4_	80.0 ± 2.0^b^	39.3 ± 1.5^b^	47.0 ± 2.0^c^	4.43 ± 0.20^c^	38.3 ± 1.5^c^
SCEE (300 mg/kg)	95.0 ± 2.6^a^	53.0 ± 3.6^a^	47.3 ± 2.5^c^	3.26 ± 0.30^d^	36.9 ± 1.6^c^

Values expressed as means ± SD. Means ± SD with different superscript letter within the column indicate significant difference (*P* < 0.05)

Absolute kidney weight of rats of various treated groups was calculated as shown in Table 7. CCl_4_ treated group showed a marked increase in absolute kidney weight when compared with the control group (*P* < 0.05). Our results of increase in kidney weight are in line with the reports of Adewole et al. [8]. However, Manjrekar et al. [36] observed a reduction in kidney mass after CCl_4_ treatments. This result may be due to the low dose and short duration of the exposure to CCl_4_.

**Table 7:** Effect of CCL_4_ treatment on kidney weight

Group	Kidney weight (g)
Control	2.2 ± 0.1^b^
Vehicle control	2.2 ± 0.2^b^
CCl_4_ (1 mL/kg)	3.5 ± 0.3^a^
Silymarin + CCl_4_	2.4 ± 0.2^b^
SCEE (150 mg/kg) + CCl_4_	2.5 ± 0.1^b^
SCEE (300 mg/kg) + CCl_4_	2.3 ± 0.2^b^
SCEE (300 mg/kg)	2.4 ± 0.3^b^

Effect of SCEE on kidney tissue protein, TBARS, H_2_O_2_ and nitrite content is given in Table 8. The concentration of total protein after 2 months CCl_4_ administration decreased significantly in renal tissues. SCEE co-treatment protected its level when compared to CCl_4_ treated group. However, its concentration remained significantly lower than that of the control group. The level of TBARS is measured to estimate lipid peroxidation. Its level was observed significantly higher in CCl_4_ treated group. SCEE co-treated groups showed dose-dependent protection against lipid peroxidation and showed equal effect to that of the reference drug silymarin treated group. The concentration of H_2_O_2_ and nitrite also showed alterations and significant rise was observed in the CCl_4_ treated group. The SCEE co-administration with CCl_4_ showed significant effect and hinders the rise of H_2_O_2_ and nitrite content dose dependently. The level of H_2_O_2_ in SCEE 300 mg/kg b.w. dose group was comparable with that of silymarin treated group but nitrite content was significantly higher than silymarin treated group.Oxidative stress caused by CCl_4_ can induce cell damages and local endemic conditions which lead to vasoconstriction and in this manner mounting the nitrite substance in urine and serum [37]. Co-treatment of SCEE appreciably reduced the nitrite substances in serum. Comparable findings were observed by MR Khan and D Ahmed [1].

**Table 8:** Effect of different treatments of *S. cordata*“ethyl acetate fraction (SCEE)” on kidney tissue protein, TBARS, H_2_O_2_ and nitrite content

Group	Protein (μg/mg tissue)	TBARS (nM/min/mg protein)	H_2_O_2_ (nM/min/mg tissue	Nitrite content(μM/mL)
Control	3.1 ± 0.1^a^	1.3 ± 0.2^d^	3.7 ± 0.2^d^	45.6 ± 2.5^e^
Vehicle control	2.9 ± 0.2^a^	1.4 ± 0.2^d^	3.9 ± 0.1^d^	51.3 ± 3.1^d^
CCl_4_ (1 mL/kg)	1.3 ± 0.2^c^	3.7 ± 0.4^a^	9.5 ± 0.1^a^	95.3 ± 1.2^a^
Silymarin + CCl_4_	2.8 ± 0.2^a^	1.8 ± 0.2^cd^	4.9 ± 0.1^c^	54.0 ± 1.0^d^
SCEE (150 mg/kg) + CCl_4_	1.5 ± 0.1^bc^	2.7 ± 0.1^b^	6.8 ± 0.3^b^	89.0 ± 2.0^b^
SCEE (300 mg/kg) + CCl_4_	1.8 ± 0.1^b^	2.3 ± 0.2^bc^	5.1 ± 0.1^c^	63.0 ± 1.0^c^
SCEE (300 mg/kg)	2.8 ± 0.1^a^	1.6 ± 0.2^cd^	3.9 ± 0.4^d^	52.0 ± 0.0^d^

Values expressed as means ± SD. Means ± SD with different superscript letter within the column indicate significant difference (*P* < 0.05)

Antioxidant enzymes system in the body plays an important role in defense against oxidative stress of free radicals and toxic chemicals. CCl_4_ induce oxidative stress and results in the decrease of antioxidant enzymes (Table 9). The activity level of CAT in renal tissues was decreased significantly after administration of CCl_4_ treatment in CCl_4_ treated groups. Co-administration of SCEE showed a significant effect and prevented the decrease of CAT dose dependently. A significant difference was observed between silymarin and SCEE co-treated group’s profile of CAT. A similar change was observed in alteration of tissue profile of POD, SOD, GST, GSH, GP_X_ and GR at a lower dose of SCEE (150 mg/kg bw). However, an equal level for these enzymes was recorded at a higher dose of SCEE (300 mg/kg bw).

**Table 9:** Effect of different treatments of *S. cordata*“ethyl acetate fraction (SCEE)” on kidney tissues antioxidant enzymes profile

Group	CAT (U/min)	POD (U/min)	SOD (U/mg protein)	GST nM/min/mg protein)	GSH (μM/g tissue)	GPx (nM/min/mg protein)	GR (Nm/min/mg protein)
Control	5.6 ± 0.2^a^	9.0 ± 0.1^a^	2.8 ± 0.1^a^	99.5 ± 2.8^a^	29.3 ± 2.5^a^	81.4 ± 2.0^a^	209 ± 5.3^a^
Vehicle control	4.9 ± 0.1^a^	8.7 ± 0.1^a^	2.5 ± 0.0^a^	97.8 ± 2.2^a^	25.7 ± 2.1^a^	78.8 ± 2.1^a^	189 ± 6.2^a^
CCl_4_ (1 mL/kg)	1.1 ± 0.1^d^	3.0 ± 0.2^c^	0.7 ± 0.1^c^	24.5 ± 1.0^c^	11.3 ± 2.5^c^	38.8 ± 1.0^c^	67.4 ± 4.2^c^
Silymerine + CCl4	5.1 ± 0.1^a^	9.2 ± 0.2^a^	2.7 ± 0.2^a^	102 ± 3.2^a^	27.3 ± 3.5^a^	80.4 ± 3.0^a^	198 ± 3.2^a^
SCEE(150 mg/kg) + CCl_4_	2.4 ± 0.1^c^	5.9 ± 0.4^b^	1.6 ± 0.3^b^	77.8 ± 1.0^b^	17.7 ± 3.1^bc^	61.5 ± 3.2^b^	99.4 ± 1.4^b^
SCEE(300 mg/kg) + CCl_4_	4.2 ± 0.2^b^	8.4 ± 0.7^a^	2.5 ± 0.1^a^	89.0 ± 1.0^a^	24.0 ± 2.7^ab^	77.5 ± 2.5^a^	187 ± 2.2^a^
SCEE(300 mg/kg) alone	5.3 ± 0.3^a^	8.9 ± 0.8^a^	2.6 ± 0.4^a^	92.0 ± 0.8^a^	31.0 ± 2.7^a^	79.9 ± 1.8^a^	199 ± 5.1^a^

Values expressed as means ± SD. Means ± SD with different superscript letter within the column indicate significant difference (*P* < 0.05)

**Fig. 1:**
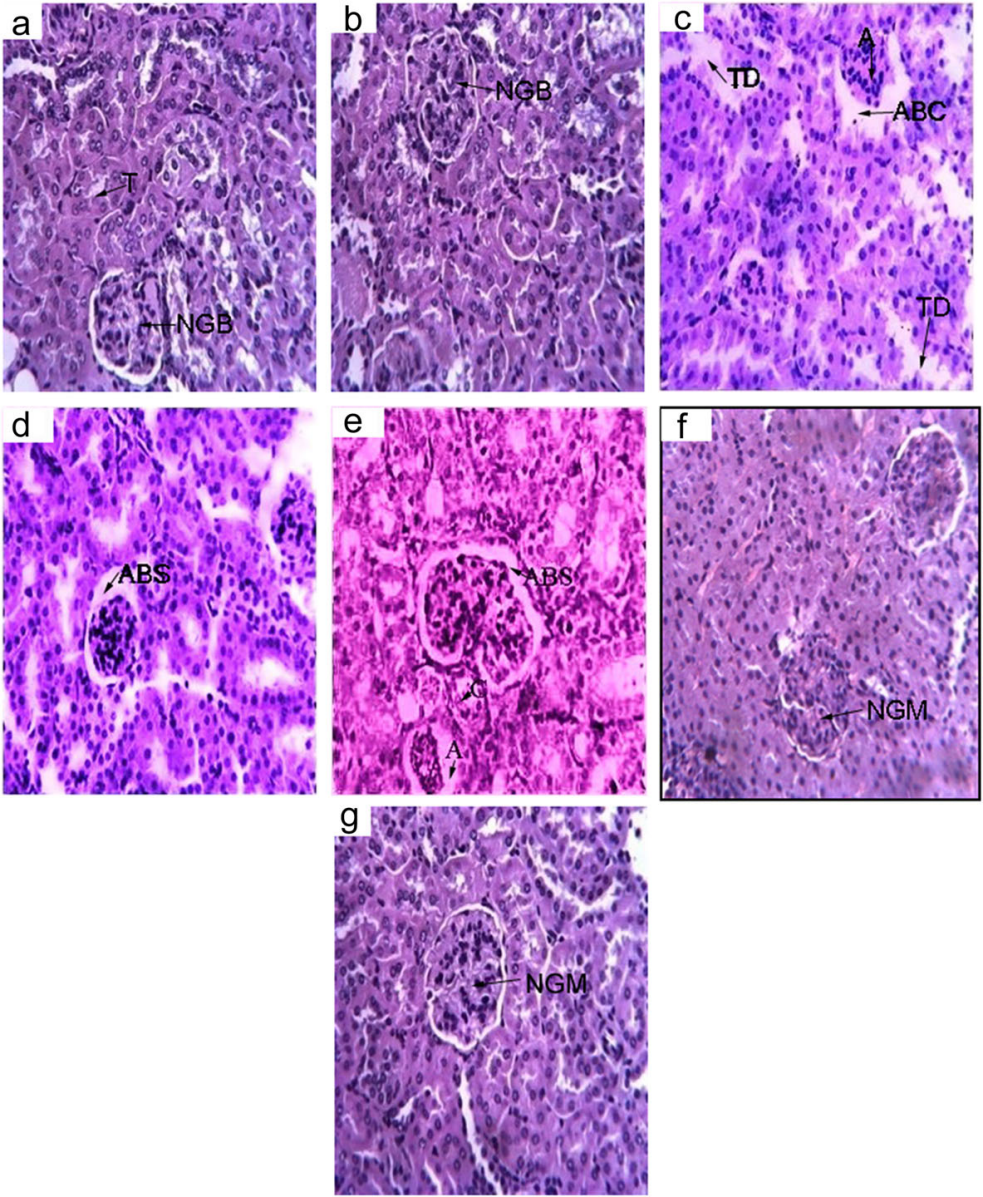
Microphotographs of kidney histology of different groups after *S. cordata*“ethyl acetate fraction (SCEE)” treatments (H & E staining). **1a. ** Control group; **1b. **Negative control group; **1c. **CCl_4_ treated control group; **1d. **CCl_4_ + Silymerin 100 mg/kg treated group; **1e. **CCl_4_ + SCEE 150 mg/kg treated group; **1f. **CCl_4_ + SCEE 300 mg/kg treated group; **1g. **Only SCEE 300 mg/kg treated group. T-Normal tubules, NGS-Normal Glomerulus and Bowmens capsule, C-congestion, TD-tubule distruction, A-atrophy, ABS-alteration in bowmen’s space

Figure 1 illustrates histoarchitecture alteration of the kidney in different groups of SCEE treatment. Kidney histology was performed with H & E stain to confirm the modulatory role of the plant extract. Intraperitoneal injection of CCl_4_ for the alternate day for 2 months induced severe destruction in the cortical region of the kidney in comparison to the medullary region. Glomerular atrophy was observed in different forms by means of disappearance and dilation of bowmen’s capsule, degeneration and dilation of renal tubules in cortical region, blood congestion in the capillary loops and inflammatory cells infiltration as shown in CCl_4_ treated group (Figure 1c). Control group showed normal glomerular structure encapsulated in bowmen’s capsule, normal tubule and afferent tubules (Fig. 1a-1b). Dose-dependent protection was observed with the treatment of SCEE. Atrophy and mild altered bowmen’s capsule space were observed at a low dose (Fig. 1e). However at the higher dose SCEE protected the CCl_4_ for inducing alterations and no histoarechitetural change was observe. (Fig. 1f). SCEE, when treated alone, showed no alteration (Fig. 1e). CCl_4_ treatment mediated lipid peroxidation of lipid structures of renal tissues, causing subcellular injuries as illustrated in histopathological examination. In the present study, the kidneys of CCl_4_ treated animals have showed morphological changes, for example, glomerular and tubular degeneration, interstitial fibrosis and interstitial mononuclear cell infiltration. The vasoconstriction actuated by CCl_4_ produces an ischemic nature’s domain, which expedites various cell damages, for example, decay in membrane integrity. Significant histological alterations are obvious in the external cortex and internal medullary section of the kidneys. The extreme alterations were not marked in the animals co-treated SCEE recommending the defensive impacts of SCEE in weakening CCl_4_ actuated morphological progressions. Comparable histopathological progressions were noted by RA Khan, MR Khan, S Sahreen and J Bokhari [38] in renal tissue of rats treated with CCl_4_ and these histopathological progressions were vanished in rats treated *Sonchus asper* extract. Tubular epithelial cells changes incorporating vacuolization, atrophy and finally separation of epithelial cells, showed tubular necrosis in kidneys of rats treated with CCl_4_. Comparable histopathological markers were likewise recorded in different studies after a long introduction to CCl_4_ [39]. It is accepted that with these histopathological progressions the limit of tubular osmosis might have been changed, therefore achieving functional over-burden of nephrons with resulting renal malfunctioning [37].

## Conclusion

These results suggest that supplementation of SCEE resulted in nephroprotection against the oxidative damage induced with CCl_4_ in rat. It was shown that SCEE has protective effect on urinary and serum profile and histopathological changes in a dose dependent manner. The protective effect of SCEE against CCl_4_-induced toxicity could be attributed to its antioxidant capacity.
